# Artificial Intelligence-Integrated Virtual Reality in Mental Health Care: A Scoping Review of Evidence, Clinical Applications, and Future Directions

**DOI:** 10.3390/jcm15113993

**Published:** 2026-05-22

**Authors:** Ahmed M. Alhuwaydi

**Affiliations:** Department of Internal Medicine, Division of Psychiatry, College of Medicine, Jouf University, Sakaka 72388, Saudi Arabia; amalhuwaydi@ju.edu.sa; Tel.: +966-56-144-9444

**Keywords:** artificial intelligence, virtual reality, machine learning, anxiety, phobia, stress

## Abstract

**Background:** Mental illness constitutes one of the greatest worldwide health burdens. The use of artificial intelligence (AI) and virtual reality (VR) is becoming increasingly relevant in mental health. Nevertheless, evidence regarding their integrated application remains sparse. This scoping review identified existing evidence on AI-integrated VR in mental health care, including clinical applications, reported outcomes, and future research directions. **Methods:** The Population, Concept, and Context framework was used as the eligibility criteria. The mental health-related studies considered were original studies that addressed explicit AI integration using VR systems or workflows and had at least one outcome or clinical or implementation finding. PubMed, Scopus, Web of Science, and PsycINFO were searched to retrieve English-language studies published between January 2020 and February 2026. **Results:** The available evidence is heterogeneous, generally small, and primarily focused on feasibility or predictive modeling. The focus of applications is on the assessment or prediction of anxiety spectrum conditions, trauma and post-traumatic stress disorders, stress, and panic disorder/agoraphobia. Most of the research examines immersive VR with multimodal inputs and machine-learning-based prediction models. However, the field remains largely in an early stage, with a lack of standardization, implementation readiness, safety reporting, and real-world validation. **Conclusions:** AI-integrated VR can be considered as a promising but emerging field, and further development requires stricter, more clinically based, and implementation-focused studies that can help establish safe, effective, and scalable implementation in mental health care. Furthermore, pragmatic, multicenter research directly investigates whether AI-integrated VR has additional clinical value compared to regular VR or regular care in mental health care.

## 1. Introduction

Globally, mental health disorders continue to be a leading health determinant of disability and diminished quality of life, with severe impacts on affected individuals, their families, and the health system [1,2]. The World Health Organization (WHO) has documented that about 1 in 7 individuals worldwide suffer from mental disorders, and recent WHO fact sheets continue to highlight the huge burden and gaps in treatment capability that exist in the treatment of common mental disorders, such as anxiety disorders [2]. The latest WHO reports have also raised the issue of persistent global deficits in mental health service capacity and access and have proposed scalable and clinically significant innovations in mental health care provision [3].

Alongside these unmet needs, artificial intelligence (AI) has become a fast-growing field in mental health care, where it is used for screening support, symptom monitoring, risk prediction, digital phenotyping, and treatment support systems [4]. Recent evidence shows that AI can be used to enhance personalization, predict symptom development, and support decision-making in mental health care. Even though this field is growing, more rigorous clinical validation, implementation research, and governance structures are needed before it is adopted routinely in mental health clinical management [5,6,7]. Similarly, virtual reality (VR) has become increasingly important in mental health care due to its potential to create controlled, repeatable, and graded simulations of real-world or therapeutic scenarios that may be challenging to replicate in traditional settings. In the realm of mental health, VR has been investigated for several clinical management areas, including but not limited to exposure-based treatment, stress management, emotional regulation, and general well-being [8,9,10].

The application of AI in VR systems is thus of particular concern in mental health care. Theoretically, AI has the potential to facilitate data-driven personalization, adaptive scenario progression, intelligent virtual agents, and the prediction of distress or symptom pathways in the context of VR-based care to enhance engagement and responsiveness to individual clinical needs [11,12,13]. Simultaneously, preliminary applications of AI and VR have already demonstrated potential value in other areas of the healthcare sector, specifically in healthcare education and simulation-based training, where reviews report enhancing adaptive learning, skills acquisition, and training focused on patient safety [14,15]. These changes underscore the need to examine the current implementation of AI-integrated VR in mental health care and the direction of the field’s development.

The combination of AI and VR has a particularly strong rationale in the field of mental health care since many psychiatric symptoms are dynamic, context-dependent, and influenced by stress, vulnerability, avoidance, arousal, and comorbidity [16]. Using the stress–vulnerability approach, it is possible to implement controlled and repeatable exposure to clinically relevant stressors when using immersive VR, and AI can be used to support individualized interpretation of physiological, behavioral or symptom–response data during such exposures [11,17]. Such synergy also aligns with the digital psychiatry approaches that focus on delivering interventions through multimodal monitoring, personalization, and decision support instead of employing a one-size-fits-all approach to delivering interventions [18]. Furthermore, psychiatric comorbidity may complicate diagnosis, treatment choice, adherence, and clinical outcomes, which supports the need to integrate adaptive models of assessment and care [16].

Although the research on AI and VR, as well as AI-integrated VR in mental health care, has gained more attention, and the interest in combining them has only grown, the evidence on AI-integrated VR in mental health care seems to be scattered in terms of conditions and study design, technological strategy, and areas of outcomes. Current reviews usually cover VR for specific mental health issues or AI in mental health, in general, but do not specifically address the implementation of AI-integrated VR within mental health care practices [5,8,9]. Moreover, terms and definitions vary across fields, and research might focus on feasibility, utility, or even implementation improvements rather than clinical improvements, making it hard to determine the current level of evidence, all possible clinical applications, and the most significant potential gaps in future research. This supports our use of a scoping review rather than a narrowly focused systematic review, as our goal was to map heterogeneous evidence, clarify application categories, and identify implementation-relevant gaps that may inform future clinical evaluation and decision-making. Therefore, this scoping review aims to provide an overview of updated and current evidence on AI-integrated VR in mental health care. Furthermore, this review specifically focused on clinical applications, reported outcomes, and future directions for research and implementation.

## 2. Materials and Methods

### 2.1. Study Design and Reporting Guidelines

The present scoping review is registered in the open-source framework (registration ID: 10.17605/OSF.IO/PU5ZH), adheres to the guidelines of scoping reviews, and is reported in accordance with the Preferred Reporting Items for Systematic Reviews and Meta Analyses extension for Scoping Reviews (PRISMA ScR) [19,20]. The PRISMA-ScR checklist is provided in Supplementary Materials (Table S1).

### 2.2. Eligibility Criteria

Eligibility criteria were developed using the Population, Concept, and Context framework [21,22] and included original research studies involving human participants in mental health-related populations or settings. Eligible studies examined the use of AI integrated with VR in mental health care or closely related psychological, affective, stress-related, or neurocognitive care contexts. In this review, both immersive and non-immersive formats of VR were considered, including head-mounted display systems and screen-based virtual environments. AI integration was defined as the use of explicit AI or machine learning (ML)-based methods (e.g., predictive modeling, adaptive personalization using data-driven algorithms, intelligent agents, and related computational approaches) within the VR system or its therapeutic workflow. For eligibility, AI integration was considered present only when the VR system or VR-derived data were linked to an explicit AI/ML-based function, such as automated classification, prediction, adaptive personalization, decision support, intelligent agent interaction, or real-time feedback. Conventional signal acquisition or feature extraction alone was not considered sufficient unless it was followed by an AI/ML-based analytic or adaptive component.

Furthermore, original studies that reported mental health-related applications, such as assessment support, treatment delivery, symptom management, monitoring, or rehabilitation, were included. For conceptual clarity, mental health-related applications were considered to include direct psychiatric or mental health conditions as well as adjacent psychological, affective, stress-related, or neurocognitive applications when the study reported a mental health-relevant outcome, such as anxiety, stress, emotional response, psychological distress, cognitive functioning, rehabilitation, acceptability, or feasibility. Therefore, studies involving subjective cognitive decline, biophilic virtual environments, or neurocognitive rehabilitation were included only when they used an explicit AI/ML component linked to VR and reported a mental health-relevant or adjacent psychological/neurocognitive outcome. These studies were included to map the broader boundary of AI-integrated VR applications relevant to mental health care but were not interpreted as direct evidence of psychiatric diagnosis or treatment effectiveness. Furthermore, at least one relevant outcome or implementation-related finding had to be present in the included studies. The present review excluded review articles, meta-analyses, editorials, commentaries, conference abstracts, study protocols without results, theses, studies with non-human subjects, gray literature, and purely technical or engineering studies that did not include human participants or mental health-related application data.

### 2.3. Information Sources and Search Strategy

This review was limited to studies published in English from 1 January 2020 to 28 February 2026. The final search was completed on 10 March 2026. This period was chosen to prioritize contemporary evidence with greater clinical relevance, given rapid advances in AI methods, the wider availability of VR technologies, and the increasing translation of digital mental health tools into applied clinical and implementation contexts. The search was conducted across major databases, such as PubMed/MEDLINE, Scopus, Web of Science, and PsycINFO. Additionally, a list of references relevant to this review was retrieved by screening the reference lists of the primary studies as per the eligibility criteria. The primary aim of the search design was to identify the review’s three core concepts: AI, VR, and mental health care. We used Boolean operators, either individually or together, that were related to AI (for example, “artificial intelligence”, “machine learning”, “deep learning”, “predictive model”, “intelligent agent”); virtual reality (for example, “virtual reality”, “immersive environment”, “virtual environment”); and mental health conditions or care contexts (for example, “mental health”, “psychiatry”, “psychology”, “anxiety”, “depression”, “stress”, “post-traumatic stress disorders [PTSD]”, “phobia”). The complete database-specific search strategies for PubMed/MEDLINE, Scopus, Web of Science, and PsycINFO, including exact Boolean search strings, date limits, language limits, and applied filters, are provided in Supplementary Files (Table S2).

### 2.4. Data Extraction and Data Synthesis

**Figure 1:**
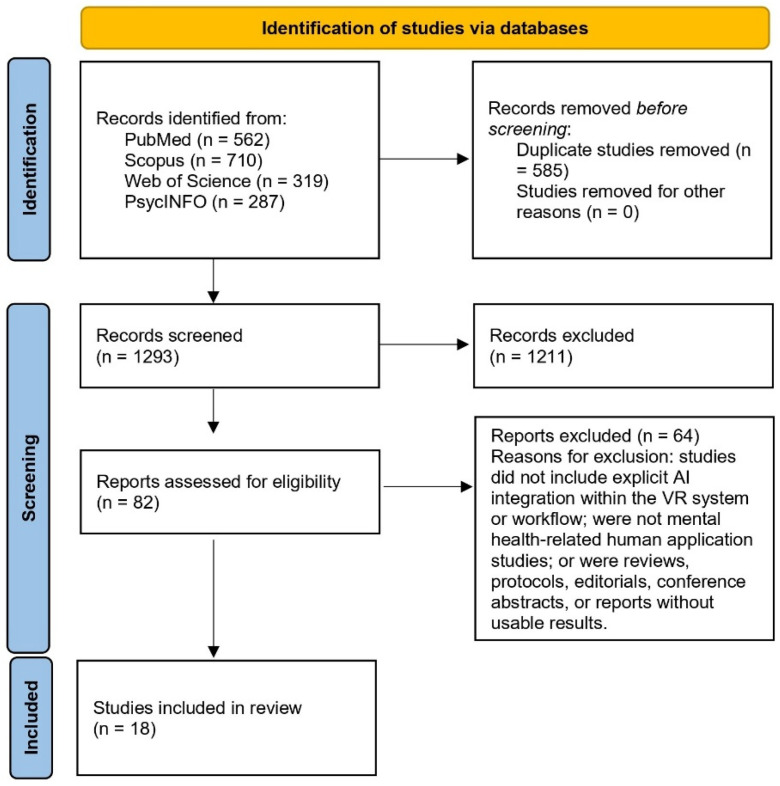
Flow diagram of study selection process.

The study selection process is depicted in Figure 1 as a PRISMA-style flow diagram adapted for scoping reviews. Study screening and data extraction were performed independently by two reviewers using predefined eligibility criteria and structured extraction fields. Any discrepancies were discussed and resolved with input from a third reviewer (please see the Acknowledgements Section for more details). Data extraction followed a structured process to include bibliographic details, country, study design, population characteristics, mental health condition or target symptom domain, VR format (immersive or non-immersive), AI component and its role, clinical application, outcomes assessed, key findings, and implementation or feasibility observations. Considering the nature of the review, no pooled effects were estimated, and no meta-analysis was conducted. The data synthesis is presented under several key themes and patterns. The extracted data were narratively charted using predefined fields matching Table 1, including study characteristics, condition/target domain with population type, VR format, AI method(s) with role/category including whether the AI component was embedded/real-time or applied offline/post hoc, key findings, and outcome type, and were then grouped into recurring themes related to clinical application, AI/VR approach, reported outcomes, feasibility, and implementation considerations. The included studies were therefore interpreted according to the degree of AI-VR integration, distinguishing embedded or real-time AI-supported VR applications from studies using offline/post hoc AI analysis of VR-derived data.

## 3. Results

To avoid overlap, the results are presented under four areas: study characteristics, clinical applications, technical and clinical outcomes, and feasibility and implementation outcomes.

### 3.1. Characteristics of Included Studies

The group of eligible studies that integrated AI/ML with VR for mental health applications was small, heterogeneous, and primarily focused on feasibility and predictive modeling. The studies focusing on outcome-focused randomized trials were limited. According to the study selection process (Figure 1), we found 18 eligible studies (on the basis of the predefined eligibility criteria) (Table 1). Among the critical domains studied were anxiety disorders (social anxiety disorder, panic disorder/agoraphobia); the detection of anxiety arousal in speech before an audience; biofeedback; and the prediction of PTSD status, especially in military personnel.

The original research included primarily focused on immersive/hybrid VR exposure conditions with multi-sensor (AI) classification/prediction of symptom severity, therapy response, or diagnosis (e.g., using AUROC to forecast sympathetic behavior; accuracy/F1 to forecast response; or the Gaussian process to classify PTSD). The most common study setting was South Korea (6/18 studies) [26,30,33,34,35,38], followed by China (2/18) [29,39]. One study each was reported from Romania [23], Spain [24], Poland [25], Canada [27], the United Kingdom [28], Italy [31], and Israel [37]. Ireland and Romania were jointly represented in one multinational study (Ireland/Romania) [32]. In addition, there were two other multinational studies, one from Saudi Arabia/Pakistan [40] and one from France/the USA [36]. Overall, the evidence base appears geographically concentrated, with a notable predominance of studies from South Korea and relatively limited representation from other regions (according to the reported affiliations and recruitment/ethics statements in the primary research studies).

### 3.2. Clinical Applications of AI-Integrated VR

For interpretive clarity, the findings were organized around four practical application domains: assessment/screening, prediction/monitoring, treatment augmentation through adaptive or biofeedback-supported VR, and usability/implementation evidence. These domains were used to organize this section’s structure. The review identified that the role of AI-integrated VR has been investigated in the treatment of a range of mental health conditions. For example, a study by Petrescu et al. exposed subjects with height phobia to a VR acrophobia scenario, integrating HR and EDA to detect anxiety [23]. Using ML, Chun et al. (2022) applied repeated VR social situations in SAD patients, measuring heart rate and galvanic skin response (GSR), monitoring to support anxiety symptom and VR sickness prediction [26]. Together, these studies represent diagnostic or symptom-classification applications, but the evidence remains mainly technical and provides proof of concept rather than confirmatory clinical diagnostic evidence.

In a study by Cheng et al. (2023), a VR experience in which a task was completed at a height was created and limb motion sensors were used to distinguish subjects with a fear of heights vs. control subjects via ML [29]. AI-integrated VR methods have also been applied in PTSD treatment. For example, a Pellegrin et al. (2025) used physiological habituation responses in an immersive VR setting for ML-based PTSD classification [36]. Likewise, in 2025, Shin et al. compared an immersive guided-imagery VR-based stabilization intervention with an app for COVID-19 survivors with PTSD symptoms. They discovered that the model was practical in detecting post-traumatic stress symptoms and associated comorbid conditions [38]. These PTSD-related studies are better interpreted as prediction and stratification studies, where AI-integrated VR may help identify symptom patterns or likely responses, although their clinical utility requires further validation.

Some authors attempted VR and AI integration for training and cognitive rehabilitation. A study from 2022 by Cuesta et al. immersed older adults with cognitive decline in two VR environments (a train and a music theater) augmented by an EEG-based “intelligent agent” that adapts to the subject’s emotional state. Their study results showed favorable feasibility and tolerability. Nonetheless, the authors suggested a larger-scale randomized clinical trial to confirm its efficacy [27]. Stasolla and Di Gioia (2023) employed a mixed RL and VR task to evaluate subjective cognitive decline and concentrate on usability for both patients and the caregivers [31]. These studies fall within adaptive rehabilitation and usability-oriented applications, with relevance to broader neurocognitive and psychological care rather than providing direct evidence of psychiatric treatment effectiveness. Mevlevioglu et al. (2024) [32] subjected older adults to the VR Stroop stress test and evaluated the level of anxiety with wearable biosignals (an XGBoost model). Their convolutional neural network (CNN) demonstrated 75% accuracy in detecting anxiety levels.

A number of studies were geared towards diagnostic or predictive implementation. Jung HW et al. (2025) subjected patients with panic disorder and agoraphobia to VR tasks and applied skin conductance response (SCR) with ML to differentiate them from controls [34]. Park J-H et al. (2025) utilized patients with social anxiety in VR public speaking activities and employed mixed physiological features to anticipate the symptom severity, as well as the results of the treatment [33]. Wu et al. (2025) showed adolescents a stressor VR test, analyzed multimodal data, and used ML (SVM) to filter depression status [39]. Zisquit et al. (2025) tested a guided VR self-talk CBT session for patients with OCD with the help of an AI chatbot and qualitatively evaluated the usability of the session [37]. This group of studies highlights the expanding use of AI-integrated VR for screening, prediction, monitoring, and guided self-reflection, but most findings remain at the early stage and require replication in larger clinical samples. A multimodal VR-based behavioral intervention proposed by Mohamed et al. (2025) uses digital interventions (including VR/AI) in mental health in the Arab region [40]. According to their study’s findings, the average heart rate decreased by 16.7% between sessions and the completion time was lowered by 21.4% [40]. This study provides preliminary evidence for adaptive interventions, although its single-arm design limits conclusions about comparative clinical effectiveness.

### 3.3. Technical and Clinical Outcomes

The outcomes were usually in terms of algorithmic precision or symptom transformation. The best accuracy for anxiety classification was achieved with the combination of heart rate and EDA characteristics during VR exposure (Petrescu et al., 2020) [23]. Kaminska et al. (2021) achieved a maximum accuracy of about 96.4% in classifying three levels of stress from EEGs during VR activity [25]. Combining seven heart rate and electrodermal activity parameters in the time and frequency domains produced the best classification accuracy [23]. Perez-Valero et al. (2021) demonstrated that regression models could closely track self-reported stress changes throughout a VR relaxation session [24]. Chun et al. (2022) forecasted the subdomain scores of anxiety (Internalized Shame Scale and Post-Event Rumination Scale) with F1 to a maximum of 0.842 and forecasted VR sickness with F1 = 0.706 [26]. These findings show promising technical performance for psychophysiological classification and prediction, but they should not be interpreted as evidence of routine clinical usefulness without external validation. The study by Rahman et al. (2023) presented the following example of a working pipeline: in their framework, adaptive biofeedback controlled arousal by providing real-time heart rate and EEG-laterality feedback to the user when exposed to VR [28].

A recent study by Cheng et al. in 2023 also indicated high classification accuracy (above 90%) between acrophobic and non-phobic movement patterns using an 80-feature ensemble model [29]. Jung et al. (2023) determined that biophilic VR rooms were distinguishable compared to controls with more than 90% EEG classification accuracy, centering on a frontal alpha power biophilic design with more low-frequency (relaxation) EEG and a positive impact [30]. The results of Mevlevioglu et al. (2024) indicated approximately 75% accuracy in classifying anxiety using real-time EEG/heart rate variability during VR training [32]. The high classification accuracies reported in these small experimental studies indicate feasibility, but the clinical meaning of these outputs remains uncertain because outcome measures and validation approaches varied across studies. Jung HW et al. (2025) were able to achieve classification performance through using physiological VR responses to classify patients with panic/agoraphobia and controls [34]. Cuesta et al. (2022) indicated that VR was tolerated among their participants; qualitative feedback indicated that the VR-EEG agent was involved [27]. Stasolla (2023) discovered that MNI patients and their caregivers could use and find RL+VR tasks [31]. The study by Zisquit et al. (2025) reported strong interest and involvement from the participants in the use of the VR module guided by AI, but no clinical results have been reported so far [37]. In contrast, these studies mainly provide usability and acceptability evidence, which is useful for implementation planning but weaker than evidence of clinical effectiveness. Across these categories, most reported outcomes represented technical performance, feasibility, or early predictive validity rather than direct evidence of clinical effectiveness. High classification accuracy or model performance in small, single-site datasets should therefore be interpreted cautiously, particularly where external validation, long-term outcomes, and real-world clinical workflow testing were limited.

### 3.4. Feasibility and Implementation Outcomes

The reviewed studies also reported practical considerations. Most interventional studies used immersive head-mounted displays (HMDs) (e.g., Oculus) with concurrent physiological sensors (EEG, ECG (electrocardiography), GSR (galvanic skin response), etc.). This combination was often justified as a way to capture both subjective immersion effects and objective physiological signals that could be used for classification, prediction, or adaptive feedback. For example, Perez-Valero (2021) utilized VR headsets plus EEG to collect real-time biosignals such as stress–relaxation experiences [24]. Adaptive and feedback features were implemented: Rahman (2023) was able to combine off-the-shelf EEG (Emotive EPOC) and heart rate variability systems with Oculus to apply biofeedback in VR exposure therapy (VRET) [28]. Cuesta (2022) focused on the ease of use: their VR exercises lasted 5–10 min, and they reported low cybersickness rates [27]. The majority of studies reported high acceptability. According to Stasolla (2023), the RL-VR was rated positively for usability by both patients and caregivers [31]. Some feasibility frameworks (Rahman, Cuesta, Stasolla) emphasized the need to simplify standard sensor calibration and ML pipelines [27,28,31]. Although major technical issues were not consistently reported, scalability and clinical readiness remain uncertain because most studies were small, experimental, and not tested in routine clinical settings or evaluated for cost, training needs, data governance, and workflow integration.

In observational/predictive studies, feasibility focused on data acquisition and sample size. Several studies have smaller sample sizes and dropouts due to several reasons, including those relating to the technology used. For example, in Park et al.’s (2024) study, only smaller samples (32 participants) were included at the initial stages [33]. However, the data for seven participants were deleted from the analysis due to sensor malfunctions. In the studies covered, feasibility and implementation lessons periodically reiterated the feasibility of pragmatic tradeoffs of immersive delivery, sensor performance, and the cost of executing AI pipelines in realistic or realistic-like settings. Although off-the-shelf devices and brief VR sessions may support feasibility, steady signal quality and streamlined calibration, cost, staff training, data governance, and workflow integration are the major factors that determine the success of implementation.

## 4. Discussion

AI-integrated VR in mental health care remains largely exploratory, with promising early proof-of-concept findings but limited evidence of confirmed clinical effectiveness. In the studies considered, the most apparent signal seems to be in cases of anxiety spectrum application and trauma-related usage, where the context of exposure is controlled and repeatable and measurable AI components can be used to allow personalization and adaptive difficulty, the interpretation of physiological signals, and the prediction of responses. This is consistent with the wider evidence that VR-based interventions are the most developed in anxiety-related conditions and that digital psychiatry is becoming more multimodal in terms of integrating multimodal approaches to develop care and substitute clinicians [17,18,41,42]. Overall, the evidence across diagnostic/screening, predictive, adaptive, and usability-focused applications remain mainly early proof-of-concept. Although several studies have reported promising technical performance, often from small samples, offline/post hoc ML analyses, and limited external validation, stronger comparative, externally validated, and clinically meaningful evidence is still needed to distinguish technical model performance from clinical effectiveness.

One of the main implications of these outcomes is that AI-enhanced VR may be most useful when applied to a specific clinical bottleneck rather than functioning as a fully autonomous treatment system. Practically, the studies presented herein imply value in at least three functions such as enhanced assessment and phenotyping, treatment augmentation (especially adaptive exposure or skills training), and monitoring and feedback loops (including real-time tailoring or post-session analytics). This assistive and augmentative framing is more in line with the existing evidence on digital psychiatry and, in part, the reason why feasibility and acceptability may be greater when clinician supervision is clear [4,18,43]. The current evidence should therefore be interpreted as demonstrating how AI can be incorporated into VR-based mental health workflows, rather than proving that AI-integrated VR is clinically superior to standard VR. At this stage, the main contribution of AI appears to be in classification, prediction, personalization, or feedback support [11,44], while its incremental therapeutic value remains to be tested in direct comparative studies against standard VR or usual care [8,9,10].

Another implication that emerges as significant is that apparent clinical promise should not be equated with readiness to scale. AI- integrated VR interventions take the risks of two sophisticated technologies: immersive systems (hardware comfort, cybersickness, sensory overload, usability burden) and AI-driven decision support (bias, transparency, overfitting, dataset shift, poor external validation). Algorithmic bias is also a concern when models developed using small or non-representative samples are applied to different clinical, cultural, or demographic groups [45,46,47]. Poor adverse-effect reporting and underreporting of symptoms such as cybersickness or clinical worsening have already been noted as a problem in previous reviews in VR psychiatry and directly influence the interpretation of feasibility, adherence, and safety. In psychiatric populations, this is especially important because immersive or adaptive VR exposure may provoke distress, dissociation, symptom worsening, or emotional overload in vulnerable users [17,48,49]. Similarly, it has been repeatedly demonstrated in the literature on AI in mental health care that high in-sample performance does not always carry over to other settings when models are not externally validated or when workflow and population differences are not taken into account [45,46,47]. In the case of AI-based VR in mental health, these concerns are heightened because the effects depend on the quality of the therapeutic alliance, immersion, the relevance of the scenarios to the culture, and the variability in user states during sessions [48,50].

The articles also pose another conceptual argument: efficacy in controlled environments is just one aspect of clinical value. Acceptability, engagement, dropout patterns, burden on therapist training, hardware logistics, and services fit in mental health may be the key contributors to real-life success alongside symptom reduction [48,51]. This is also in line with the frameworks of implementation science, which separate clinical outcomes from implementation outcomes such as acceptability, adoption, feasibility, fidelity, penetration, and sustainability. The findings related to the feasibility of the studies in this regard are not secondary observations; they are core factors that could influence whether AI- integrated VR interventions can leave the research unit and enter routine psychiatric and psychological services [51,52].

Other studies may have had the advantage of using AI as a background analysis signal (such as response stratification or stimulus modification), but this does not imply that the intervention should be treated as a free therapeutic agent [53,54]. Accordingly, clinician oversight, clear escalation pathways, and human responsibility for clinical decisions should remain central in psychiatric AI-integrated VR applications [44,52,54]. The existing ethics and governance recommendations for AI in health care focus on human control, transparency, risk proportion, and responsibility, and these factors are particularly essential in vulnerable psychiatric groups [44,52,54]. AI- integrated VR systems may also process sensitive physiological, movement, speech, behavioral, and symptom-response data; therefore, privacy protection, secure storage, and transparent data governance are essential [53]. This is especially relevant in the context of PTSD, psychotic symptomatic effects, acute anxiety, and populations at risk of developing dissociation or distress escalation, as poorly calibrated automation might cause harm even with technically advanced systems [45,48].

Methodologically, the field would benefit from tighter alignment with emerging AI and digital intervention reporting standards. The heterogeneity across AI-integrated VR mental health studies limits cross-study synthesis and may inflate confidence in early findings. Thus, guidelines like CONSORT-AI, SPIRIT-AI, DECIDE-AI, TRIPOD+AI, and PROBAST+AI can provide a convenient framework for enhancing reproducibility, clarifying human–AI interactions, recording input data quality, and limiting the effects of risks of bias in predictive components [44,47,51,52,55]. Similarly, VR-specific recommendations on trial methodology are quite applicable to the standardization of intervention dose, description of hardware, usability reporting, and meaningful clinical endpoints [56].

Finally, this review’s findings need to be interpreted in light of a rapidly evolving evidence base. Recent studies indicate that VR still has a clinical role in anxiety-related disorders, and VR research on psychosis is also on the rise, especially with regard to paranoia and social functioning [28,57,58]. Nevertheless, the particular added value of AI (as opposed to common VR provision) remains hard to pinpoint in most studies.

## 5. Identified Research Gaps and Future Research Directions

Despite encouraging progress, the current evidence base reveals several important research gaps that should guide the next phase of work in AI-integrated VR for mental health.

- There is a lack of large, representative studies: Most studies are early-phase, feasibility-oriented, or small-scale, limiting the ability to generalize to other populations, diagnoses, and settings.
- Standardization is still weak: there is significant variation in VR hardware, degree of immersion, AI techniques, input signals, and outcome measures, reducing the comparability between studies.
- Clinical effectiveness requires more evidence: There are studies demonstrating technical potential, but fewer that indicate added value for patient outcomes, clinical decision-making, efficiency, or access to care.
- Long-term results: There is limited information on durability, long-term engagement, relapse prevention, and real-world functioning.
- The readiness to implement is insufficiently studied: The practical impediments of cost, maintenance, training, workflow integration, and scalability are not always analyzed.
- The models of human oversight are poorly specified: There is a lack of evidence on clinician-in-the-loop workflow, AI output interpretation, and escalation.
- Safety and tolerability should be better reported: Adverse effects, symptom worsening, emotional overload, and disengagement must be tracked and reported systematically.
- Equity and fairness are underexplored: In future research, subgroup performance, cultural adaptability, accessibility, and the possible bias of AI and VR systems should be evaluated.
- The mechanisms of benefit are not yet well understood, it is not always clear which AI elements (e.g., personalization, adaptation, multimodal sensing) are responsible for the observed effects.
- There should be greater emphasis on translational research pathways: Future research should build on early proof-of-concept experiments toward systematic development, validation, implementation experimentation, and service composition.
- Future studies should prioritize comparative designs, including pilot RCTs, adequately powered RCTs, and pragmatic multicenter trials. They should also use standardized clinical, usability, safety, and implementation outcomes, with clearer reporting of AI components and VR delivery in line with relevant guidance such as CONSORT-AI, SPIRIT-AI, TRIPOD+AI, PROBAST+AI, and VR-specific trial recommendations.

## 6. Strengths and Limitations

The present review has several strengths. Firstly, it addresses one of the most rapidly evolving and critical health conditions that intersects with three areas (AI, VR, and mental health). Next, a scoping review design is suitable for mapping this large, heterogeneous field, encompassing various study designs, populations, AI and VR applications, and types of outcomes. Third, in addition to providing a summary of clinical applications and reported outcomes based on recent evidence (from 2020 onwards), this review also provides insights into the feasibility and implementation of the integration. These have translational potential when applied in real-world mental health settings.

However, several limitations need to be considered. Firstly, as a scoping review, this review provides a roadmap and summarizes the available evidence but does not provide a definitive estimate of effectiveness or comparative efficacy, as is commonly done in systematic reviews and meta-analyses. Secondly, this scoping review included heterogeneous studies, varied outcome methods (AI and VR integration), and varied sample sizes. Therefore, direct comparability is limited. In addition, a formal risk-of-bias assessment (an integral component of systematic reviews) is not always performed in scoping reviews, which may limit interpretation of the methodological quality of the included evidence. Importantly, the current evidence does not yet establish whether AI-integrated VR provides measurable clinical benefit beyond standard VR, because the included studies generally did not directly compare AI-integrated VR with non-AI VR or usual care. Therefore, the added value of AI should be considered a key hypothesis for future comparative studies rather than an established finding. Another limitation is that the search strategy used broad mental health terms and selected psychiatric symptom terms but did not include all disorder- or application-specific keywords. Therefore, studies indexed mainly under terms such as dementia, cognitive impairment, psychosis, schizophrenia, addiction, substance use, VRET, in virtuo, spider phobia, or arachnophobia may have been missed if they did not also include the broader search terms used. Accordingly, this review should be interpreted as a mapping of evidence retrieved through the predefined strategy, rather than an exhaustive account of all AI and VR applications across every mental disorder or neurocognitive condition. Finally, the integration of AI and VR in mental health is novel and emerging, and there is a possibility of publishing positive, novel findings, which, in turn, may lead to publication bias in the included studies.

## 7. Conclusions

This scoping review shows that AI-integrated VR is an emerging and promising but still exploratory approach in mental health care. Its current applications lie in several aspects of mental healthcare, such as assessment, prediction, classification, and adaptive support. Moreover, the available body of literature suggests that there may be potential clinical relevance for AI-integrated VR, but this should be interpreted cautiously because comparative evidence against standard VR or usual care remains limited. However, the field remains heterogeneous and largely at an early stage, with important gaps in standardization, long-term effectiveness, implementation readiness, safety reporting, and real-world validation. An important next step will be pragmatic, multicenter research that directly investigates whether AI-integrated VR has additional clinical value compared to standard VR or regular treatment in mental health care.

## Figures and Tables

**Table 1:** Characteristics of included studies on artificial intelligence-integrated virtual reality in mental health care.

Author and Year	Country	Study Type/Design	Condition/Target Domain	VR Format	AI Method(s) and Role/Category	Key Findings	Outcome Types
Petrescu L et al. (2020) [23]	Romania	Experimental feasibility study	Anxiety detection during phobia-related (heights) VR exposure; psychophysiological monitoring for therapy adaptation Population: Non-clinical/experimental participants	Immersive VR (heights/acrophobia exposure environment)	Embedded/real-time classification: Regression and classification modeling using biosignal features	VR biosignals (HR and EDA) were used to estimate/classify anxiety in a phobia-related exposure setting.	Technical performance; psychophysiological monitoring
Perez-Valero E et al. (2021) [24]	Spain	Experimental ML validation study	Stress quantification during VR stress–relaxation session Population: Non-clinical/experimental participants	Immersive/360-degree VR stress–relaxation session (hybrid VR exposure context)	Offline/post hoc prediction: Regression/predictive modeling from EEG features to estimate stress level	EEG features during VR stress–relaxation sessions were used to estimate stress, suggesting potential AI-assisted psychophysiological monitoring	Technical performance; stress-estimation outcome
Kamińska D et al. (2021) [25]	Poland	Experimental stress-classification study	Stress/anxiety-like mental stress recognition during VR stress–relaxation task Population: Non-clinical/experimental participants	Immersive interactive VR simulation with alternating stressful and relaxing scenes	Offline/post hoc classification: CNN and conventional ML classifiers on EEG features for stress level classification	EEG during VR stress–relaxation tasks enabled ML classification of stress states in healthy adults	Technical performance; stress-classification
Chun Y J et al. (2022) [26]	South Korea	Mixed-methods predictive modeling study	SAD; symptom prediction and VR sickness Population: Clinical population with social anxiety disorder	VR treatment sessions	Offline/post hoc prediction: ML predictive models using in situ autonomic physiological signals	Autonomic signals collected during VR treatment predicted specific SAD symptom profiles and VR sickness risk, suggesting possible individualized monitoring during VRET	Technical and clinical symptom-related prediction
Cuesta M et al. (2022) [27]	Canada	Feasibility study with control group	Subjective cognitive decline (emotion and cognition) Population: Adjacent neurocognitive/older-adult population	Immersive VR	Embedded/adaptive AI: EEG-based intelligent agent for adaptive content/interactions	Feasibility findings suggest VR with an EEG-informed agent may improve emotional/cognitive engagement in older adults with subjective cognitive decline	Usability/feasibility; emotional and cognitive engagement
Rahman MA et al. (2023) [28]	United Kingdom	Implementation-focused research/feasibility framework study	Public-speaking anxiety; arousal detection and biofeedback in VRET Population: Anxiety-relatedimplementation-focused participants	Immersive HMD VR (Oculus Quest 2 reported in model summary)	Embedded/real-time biofeedback: ML-based arousal detection using multimodal EEG/HRV data	Proposed and evaluated an ML-enabled, biofeedback-driven self-guided VRET framework to enhance arousal-aware adaptation for anxiety caused by public speaking	Technical feasibility; implementation-related outcome
Cheng X et al. (2023) [29]	China	Experimental ML classification study	Acrophobia-related fear response/body-movement characteristics in VR height scenes Population: Symptom-defined/non-clinical comparison group	Immersive VR height scenes	Offline/post hoc classification: ML analysis of motion features associated with acrophobia responses	Movement features in VR height scenes were classified with ML to characterize acrophobia-related responses	Technical performance; behavioral classification
Jung D et al. (2023) [30]	South Korea	Experimental EEG-ML study	Emotional response/psychological comfort in virtual hospital environments Population: Non-clinical/adjacent psychological response population	VR hospital room simulation (biophilic design conditions)	Offline/post hoc classification: ML analysis of EEG signals across VR design conditions	EEG and ML differentiated emotional/neural responses across VR hospital room design conditions	Technical performance; affective/psychological response
Stasolla F and Di Gioia M. (2023) [31]	Italy	Usability assessment study	Mild neurocognitive impairment; rehabilitation usability Population: Adjacent neurocognitive rehabilitation population	VR rehabilitation environment	Embedded/adaptive AI: RL for VR task adaptation	RL-integrated VR usability study in patients/caregivers; adjacent mental health-relevant application	Usability/feasibility
Mevlevioğlu D et al. (2024) [32]	Ireland/Romania	Experimental real-time ML classification study	Anxiety classification in VR therapy context Population: Experimental/VR therapy-context participants	VR therapy setting	Real-time classification: CNN for real-time anxiety classification using biosensors; compared with ANN/SVM/kNN/RF/XGBoost	A CNN-based biosensor pipeline enabled real-time anxiety classification in a VR therapy context and outperformed several comparator ML methods	Technical performance; real-time anxiety-classification outcome
Park J-H et al. (2025) [33]	South Korea	Predictive modeling study	Social anxiety disorder; symptom severity prediction Population: Clinical population with social anxiety disorder	VR sessions (multimodal data collection during VR)	Offline/post hoc prediction: Ensemble/tree-based ML models (RF, XGBoost, LightGBM, CatBoost)	Multimodal data from VR sessions predicted SAD symptom severity, suggesting risk stratification and monitoring in SAD care	Technical and clinical symptom-prediction
Jung HW et al. (2025) [34]	South Korea	Case–control ML diagnostic study	Panic disorder and agoraphobia diagnosis Population: Clinical population with panic disorder/agoraphobia and controls	VR exposure scenarios	Offline/post hoc classification: ML classification using physiological measures (and symptom-related data	A VR-based multimodal ML approach discriminated against panic disorder/agoraphobia cases from controls using physiological responses during exposure	Technical and clinical diagnostic-classification
Kim B-H et al. (2025) [35]	South Korea	Longitudinal predictive modeling study	Panic disorder; early treatment response prediction Population: Clinical population with panic disorder	Simulated VR assessment experiences/tool	Offline/post hoc prediction: CatBoost classifier for treatment response prediction	Simulated VR-derived data were used to predict early treatment response in panic disorder, suggesting utility for treatment planning and stratification	Clinical prediction; treatment-response estimation
Pellegrin G et al. (2025) [36]	United States/France	ML diagnostic classification study	PTSD assessment in military personnel/veterans Population: Military/veteran population with PTSD-related assessment	Immersive virtual environment	Offline/post hoc classification: Gaussian process classifier with SHAP using physiological habituation features	Physiological habituation responses in an immersive VR setting enabled ML-based PTSD classification with reported explainability analyses	Technical and clinical diagnostic-classification
Zisquit M et al. (2025) [37]	Israel	Formative qualitative study	Psychological counseling/self-reflection support Population: Mental health counseling/self-reflection participants	Immersive avatar-based VR self-talk environment	Embedded/conversational AI: LLM-enabled virtual human for guided VR self-talk	AI-integrated VR self-talk counseling was feasible/acceptable in a formative study and showed potential to support guided self-reflection	Usability/acceptability
Shin Y et al. (2025) [38]	South Korea	Prospective clinical predictive study	Post-traumatic stress symptoms; prediction of VR-based stabilization therapy efficacy Population: Clinical population with post-traumatic stress symptoms	Immersive VR-based stabilization sessions (psychiatric treatment context)	Offline/post hoc prediction: ML models using baseline clinical and multimodal/session-related features to predict treatment response	ML models predicted response to VR-based stabilization in people with post-traumatic stress symptoms	Clinical prediction; treatment-response estimation
Wu Y et al. (2025) [39]	China	Clinical ML screening study	Adolescent depression screening Population: Adolescent screening population	VR-based multimodal assessment framework	Offline/post hoc screening: ML models integrating multimodal behavioral/physiological signals for depression screening	A VR-based multimodal ML framework showed preliminarily promising performance for adolescent depression screening	Technical performance and clinical screening
Mohamed HG (2025) [40]	Saudi Arabia/Pakistan	Single-arm interventional study	Specific phobia (spider fear/arachnophobia) Population: Clinical/symptom-defined population with specific phobia	Immersive VR exposure therapy	Embedded/adaptive AI: AI-assisted adaptive control with behavioral process analysis	An AI-integrated adaptive VR exposure program showed preliminary improvement trends in spider fear across sessions	Preliminary clinical outcome; feasibility

AI, artificial intelligence; ML, machine learning; VR, virtual reality; HMD, head-mounted display; EEG, electroencephalography; HR, heart rate; EDA, electrodermal activity; HRV, heart rate variability; CNN, convolutional neural network; ANN, artificial neural network; SVM, support vector machine; kNN, k-nearest neighbors; RF, random forest; SHAP, Shapley additive explanations; PTSD, post-traumatic stress disorder; LLM, large language model; SAD, social anxiety disorder; VRET, virtual reality exposure therapy.

## Data Availability

This scoping review used data extracted from previously published studies. No new primary dataset was generated, and all data relevant to the review are included within the article.

## Supplementary Materials

The following supporting information can be downloaded at: https://www.mdpi.com/article/10.3390/jcm15113993/s1, Table S1: Preferred Reporting Items for Systematic reviews and Meta-Analyses extension for Scoping Reviews (PRISMA-ScR) Checklist; Table S2: Full database-specific search strategies.
