# Supplementary material for: Artificial Intelligence-Integrated Virtual Reality in Mental Health Care: A Scoping Review of Evidence, Clinical Applications, and Future Directions
Source: J Clin Med. 2026 May 22;15(11):3993. doi: 10.3390/jcm15113993 (PMC13257321; doi:10.3390/jcm15113993)
Supplement: Supplementary file 1 [file jcm-15-03993-s001.zip › Supplementary Table S2_Revised.docx]

**Supplementary Table S2. Full database-specific search strategies**

Final search date: [10 March 2026]. Publication period searched: 1 January 2020 to 28 February 2026. Language limit: English. Records retrieved before deduplication are reported according to the manuscript PRISMA flow diagram and should be checked against the final database export.

| **Database/source** | **Full search strategy** |
| --- | --- |
| PubMed/MEDLINE | (("Artificial Intelligence"[Mesh] OR "Machine Learning"[Mesh] OR "Deep Learning"[Mesh] OR "artificial intelligence"[tiab] OR "machine learning"[tiab] OR "deep learning"[tiab] OR "neural network*"[tiab] OR "predictive model*"[tiab] OR "classification model*"[tiab] OR "intelligent agent*"[tiab] OR "large language model*"[tiab] OR chatbot*[tiab] OR "reinforcement learning"[tiab])  AND  ("Virtual Reality"[Mesh] OR "virtual reality"[tiab] OR VR[tiab] OR "immersive virtual reality"[tiab] OR "virtual environment*"[tiab] OR "head-mounted display*"[tiab] OR HMD[tiab] OR "360-degree"[tiab])  AND  ("Mental Health"[Mesh] OR "Mental Disorders"[Mesh] OR Psychiatry[Mesh] OR "mental health"[tiab] OR psychiat*[tiab] OR psycholog*[tiab] OR anxiety[tiab] OR depression[tiab] OR depressive[tiab] OR stress[tiab] OR "post-traumatic stress disorder"[tiab] OR "posttraumatic stress disorder"[tiab] OR PTSD[tiab] OR phobia*[tiab] OR "panic disorder"[tiab] OR agoraphobia[tiab] OR "social anxiety"[tiab] OR OCD[tiab] OR "obsessive compulsive"[tiab]))  AND  ("2020/01/01"[Date - Publication] : "2026/02/28"[Date - Publication]) |
| Scopus | TITLE-ABS-KEY ( "artificial intelligence" OR "machine learning" OR "deep learning" OR "neural network*" OR "predictive model*" OR "classification model*" OR "intelligent agent*" OR "large language model*" OR chatbot* OR "reinforcement learning" )  AND  TITLE-ABS-KEY ( "virtual reality" OR VR OR "immersive virtual reality" OR "virtual environment*" OR "head-mounted display*" OR HMD OR "360-degree" )  AND  TITLE-ABS-KEY ( "mental health" OR psychiat* OR psycholog* OR anxiety OR depression OR depressive OR stress OR "post-traumatic stress disorder" OR "posttraumatic stress disorder" OR PTSD OR phobia* OR "panic disorder" OR agoraphobia OR "social anxiety" OR OCD OR "obsessive compulsive" )  AND PUBYEAR > 2019 AND PUBYEAR < 2027  AND ( LIMIT-TO ( LANGUAGE, "English" ) ) |
| Web of Science | TS = (("artificial intelligence" OR "machine learning" OR "deep learning" OR "neural network*" OR "predictive model*" OR "classification model*" OR "intelligent agent*" OR "large language model*" OR chatbot* OR "reinforcement learning")  AND  ("virtual reality" OR VR OR "immersive virtual reality" OR "virtual environment*" OR "head-mounted display*" OR HMD OR "360-degree")  AND  ("mental health" OR psychiat* OR psycholog* OR anxiety OR depression OR depressive OR stress OR "post-traumatic stress disorder" OR "posttraumatic stress disorder" OR PTSD OR phobia* OR "panic disorder" OR agoraphobia OR "social anxiety" OR OCD OR "obsessive compulsive")) |
| PsycINFO | ((DE "Artificial Intelligence" OR DE "Machine Learning" OR "artificial intelligence" OR "machine learning" OR "deep learning" OR "neural network*" OR "predictive model*" OR "classification model*" OR "intelligent agent*" OR "large language model*" OR chatbot* OR "reinforcement learning")  AND  (DE "Virtual Reality" OR "virtual reality" OR VR OR "immersive virtual reality" OR "virtual environment*" OR "head-mounted display*" OR HMD OR "360-degree")  AND  (DE "Mental Health" OR DE "Mental Disorders" OR DE "Psychiatry" OR "mental health" OR psychiat* OR psycholog* OR anxiety OR depression OR depressive OR stress OR "post-traumatic stress disorder" OR "posttraumatic stress disorder" OR PTSD OR phobia* OR "panic disorder" OR agoraphobia OR "social anxiety" OR OCD OR "obsessive compulsive")) |
| Reference-list screening | Backward reference screening was performed by manually checking the reference lists of all included studies and relevant review articles identified during full-text assessment. Any potentially eligible records identified through reference screening were de-duplicated and assessed using the same PCC eligibility criteria used for database-derived records. |

Abbreviations: AI, artificial intelligence; DE, database descriptor/thesaurus term; HMD, head-mounted display; ML, machine learning; OCD, obsessive-compulsive disorder; PCC, Population-Concept-Context; PTSD, post-traumatic stress disorder; TS, topic search; VR, virtual reality.
